# Co-encapsulation of pancreatic islets and pentoxifylline in alginate-based microcapsules with enhanced immunosuppressive effects

**DOI:** 10.1007/s40204-016-0049-3

**Published:** 2016-04-29

**Authors:** Seyedeh Azin Azadi, Ebrahim Vasheghani-Farahani, Sameereh Hashemi-Najafbabadi, Aliashraf Godini

**Affiliations:** 1 Biomedical Engineering Division, Faculty of Chemical Engineering, Tarbiat Modares University, P.O. Box 14115-143, Tehran, Iran; 2 Department of Physiology, School of Medicine, Kermanshah University of Medical Sciences, Kermanshah, Iran

**Keywords:** Pancreatic islets, Alginate, Dextran–spermine, Pentoxifylline, Encapsulation

## Abstract

Alginate-based scaffolds have received considerable attention for biomedical application because of their biocompatibility and ease of preparation. The application of alginate hydrogels for encapsulation of pancreatic islets is known as a potential treatment for type I diabetes. In this study, dextran–spermine coated microcapsules of alginate containing pancreatic islets were prepared, and then co-cultured with lymphocytes for 7 days. In addition, to prevent fibrosis and evaluating the effect of anti-inflammatory drugs, pentoxifylline was loaded in the inner layer of microcapsules. Intact and encapsulated islets in an external solution of pentoxifylline were taken as two separate controls in this study. Infrared and scanning electron microscope analyses showed polyelectrolyte complex formation between alginate and dextran–spermine. In vitro tests showed that interleukin-2 secretion from lymphocytes co-cultured with encapsulated islets containing pentoxifylline in the inner layer of microcapsules was 63.6 % lower than the corresponding value for encapsulated islets without the anti-inflammatory drug.

## Introduction

There has been an increasing interest in the biocompatible tissue engineering scaffolds with drug release capacity, while protecting cells against the immune system. Transplantation of microencapsulated cells has been proposed as a therapeutic option for a wide variety of diseases such as hypothyroidism (Chen et al. 1990), hemophilia, central nervous system insufficiencies (Bensadoun et al. 2003) and diabetes mellitus (Qi et al. 2004). During the past decades, many efforts were made to reach an implantable bioartificial pancreas. Most of these devices were based on encapsulation of pancreatic islets in microcapsules or hollow fibers (Tam et al. 2005). The classical type of multilayer microcapsules was designed by Lim and Sun (1980) for preparing bioartificial endocrine pancreas. This technology is based on the formation of polycationic membrane around a polyanionic core of sodium/calcium-alginate. Although the use of alginic acid derivatives is very common, many studies on amino acid polycations have been changing during the last 25 years (Calafiore and Basta 2014). Among different types of the alginate-polycation microcapsules, alginate–poly-l-lysine (PLL)–alginate was prepared in the early works. But in more recent studies, PLL was replaced with other polycations such as poly-l-ornithine (PLO) (Luca et al. 2001) or chitosan (Madihally and Matthew 1999). Application of PLO instead of PLL resulted in stronger microcapsules and provided better perm-selectivity (Darrabie et al. 2005). Although most of these microcapsule designs were successful with promising results, some problems were encountered, especially when these systems were interacted by cells for long term (Ponce et al. 2006). Thus, there has been a need to address other kind of polycations as well as some chemical drug such as ketoprofen to increase immunosuppression (Ricci et al. 2005).

Although microcapsules structure reduced the attack of macrophages to the implants, inflammation often arises from the insertion of foreign objects like implants into the body. This can lead to fibrous/collagenous encapsulation of the implants, with consequent failures (Goreish et al. 2004). Different studies have shown that the addition of anti-inflammatory drugs to biopolymeric scaffolds can increase the success rate of implant surgeries (Baruch et al. 2009). Pentoxifylline (PTX) is an anti-inflammatory drug that has been known to inhibit immune cells from producing cytokines such as interleukin-2 (IL-2) and TNF-α, besides prevent tissue fibrosis (Berman et al. 1992). PTX is known to exert its effects through phosphodiesterase inhibition, resulting in an increase in intracellular level of cAMP (Nagy et al. 1999). In this study, cationic dextran–spermine was synthesized by the method described in the literature (Mohammad-Taheri et al. 2012). Then, dextran–spermine as a polycation together with alginate as a polyanion was used for co-encapsulation of pancreatic islets and pentoxifylline as an immunosuppressive drug to develop a cell therapy system for possible treatment of the insulin dependent diabetes mellitus.

## Materials and methods

### Animal care and use

Animals were handled according to the standard principles of laboratory animal care; the study was approved by the local ethics committee of Tarbiat Modares University. For islet isolation, male Wistar rats, 200–250 g, and for lymphocyte tests male C57B1/6 mice (6–8 weeks) were obtained from Pasteur Institute, Tehran, Iran. These animals (rats and C57B1/6 mice) were housed in groups of three per cage, under controlled conditions of light (12 h light/dark cycles), temperature (22 ± 3 °C) with free access to food and water. All experiments were performed according to the animal welfare act (Act P.L. 99-198).

### Islets isolation

For islet isolation, the modified method of Lacy and Kostianovsky (1967) with slight further modification was used. In brief, the animals were anesthetized (60 mg/kg sodium pentobarbital i.p), laparotomized, and killed by heart incision. The pancreas was inflated through the bile duct with injection of 10 mL ice-cold Hanks’ balanced salt solution (HBSS) containing 0.5 mg/mL of collagenase P, then removed, minced with scissors, and digested for 15–17 min at 37 °C. Digestion was terminated by adding 30 mL ice-cold HBSS, and the tube was shaken for 1 min. The suspension was filtered through a 500 μm plastic mesh to discard any undigested tissue. After three washes with cold HBSS, islets were hand-picked under a stereomicroscope (Godini et al. 2014).

### Preparation of splenic lymphocytes

The spleen was obtained aseptically from male C57B1/6 mouse and washed twice with HBSS. About 4–5 mL RPMI without fetal bovine serum (FBS) was injected into the spleen, so that all cells were elicited. In the next stage, these cells were put into falcon and centrifuged at 200×*g* for 15 min at 4 °C, and then the opaque interface containing lymphocytes was mixed with RBC lysis buffer to eliminate remaining red blood cells. After washing three times with phosphate-buffered saline (PBS), lymphocytes were suspended in RPMI. Moreover, the viability of the isolated splenic lymphocytes was determined by staining with trypan blue (Gibco, Paisley, Scotland).

### Preparation of islets and drug- loaded microcapsules

Alginate with high guluronic acid was obtained from BDH (UK). This content was dissolved in sterile saline (0.9 % w/v NaCl) to obtain a solution of 2 % (w/v) concentration (Tam et al. 2011). To prepare co-encapsulated drug/islets, 2 % (w/v) alginate solution was mixed with 400 µg/mL pentoxifylline solution. The drug solution was prepared in advance by dissolving PTX in filtered PBS as solvent (Dang et al. 2013). Then, this new solution (drug solution + alginate solution) was sterilized by 0.22 µm mesh filter and stirred overnight to ensure homogenous dissolution. During the mixing period, the container (beaker) was wrapped in aluminum foil to avoid light exposure which might oxidize the drug. After islets isolation, suspension of rat islets (HBSS and 2,000 µL RPMI + 10 % FBS) was mixed with the alginate solution in the presence or absence of drug. Microcapsules containing islets or drug/islets were prepared by drop-wise addition of islets/alginate or islets/drug/alginate suspension into CaCl_2_ solution (0.1 % w/v). The droplets were generated by extrusion of above suspensions through a 22G needle at a volumetric rate of 0.15 mL/min and 6 kV voltages by syringe pump (Longer Pump, LSP01 1A/2A Single Channel Syringe Pump, China). Encapsulated islets (with or without drug) were then left in CaCl_2_ solution for 10 min for further cross linking, while reaction mixture was surrounded by ice. Then, microencapsulated islets (with or without drug) were washed with filtered saline three times. To form double-layer microcapsules, alginate microcapsules were immersed in cationic dextran–spermine solution (0.05 % w/v) in saline, that was filtered by 0.22 µm mesh filter, for 5 min (Ponce et al. 2006). Microencapsulated islets were then rinsed three times by saline. For comparison, suspensions of intact and microencapsulated islets in PTX solution were used as the control samples. The surrounding PTX media were refreshed every day over the course of 1 week with a dosage equal to that of drug co-encapsulated with islets.

### Capsules morphology

Microcapsules made of alginate/dextran–spermine were dried after preparation and then coated with gold. Scanning electron microscope (SEM) (Hitachi S-3000, Japan) was used to observe the round shape and roughness of microcapsules. Moreover, fluorescence images clearly illustrated the spherical structure of microcapsules and the way that islets were enveloped in microcapsules.

### Fourier transform infrared spectrum (FTIR)

Microcapsules were ground, mixed with KBr and pressed into tabs. To verify crosslinking of microcapsules, they were scanned in the range of 4,000–500 cm^−1^ by FTIR (Spectrum Gx, PerkinElmer, MA, USA) with a resolution of 4 cm^−1^ and transmittance peaks were identified (Thanos et al. 2006).

### Viability and functional activity of encapsulated islets

After encapsulation of islets in alginate/dextran–spermine double layer hydrogels, the viability was determined by fluorometric acridine orange (AO) (Sigma) inclusion and propidium iodide (PI) (Sigma) exclusion dyes. AO was stained only in the viable cells leading to green color and PI was stained only in the dead cells which results in red color (Nabavimanesh et al. 2015). Both viable and nonviable islets cells were classified by the simultaneous use of these dyes. To do this, 1 mL of PBS (pH 7.4) containing 0.67 µM of AO and 75 µM of PI was added to 1 mL of encapsulated and co-encapsulated islets suspension in RPMI. The viability test was carried out at room temperature for 1 min in dark points after 3, 5 and 7 days of incubation.

### Co-culturing of islets and lymphocytes

Three BALB/c mice were sterilized and three thymus were punched to obtain lymphocyte without red blood cells. About 15–25 encapsulated and co-encapsulated islets were co-cultured with 5 × 10^5^ lymphocytes in each well of a 24-well plate in 1000 µL of RPMI medium containing 10 % FBS in 95 % O_2_ and 5 % CO_2_ for 7 days at 37 °C. To determine the secretion of IL-2, 150 µL of the medium was sampled at 3, 5, and 7 days of incubation and frozen at −70 °C for subsequent measurement. After 3, 5 and 7 days of incubation, the supernatant of each well was collected and kept at −70 °C for analysis of secreted IL-2 (de Vos et al. 2002). After each measurement, the same amount of fresh medium was added into each well, to maintain the volume of culture medium constant. The secretion of mouse IL-2 was assessed by enzyme-linked immunosorbent assay (ELISA) using a commercial kit (Enzo Life Sciences, USA).

### Pentoxifylline release from microcapsules

To study the release profile of pentoxifylline from microcapsules, 20–30 microcapsules were suspended in 2,000 µL of PBS and shaken in a water bath at 80 rpm during the test. At predetermined time intervals, 1,000 µL of PBS was sampled, and replaced with the same volume of fresh PBS. The absorbance of PTX at 272 nm was measured against calibration curve to obtain the cumulative release of drug (Christova-Bagdassarian et al. 2007; Tabata 2005).

## Result and discussion

### SEM analysis

**Fig. 1:**
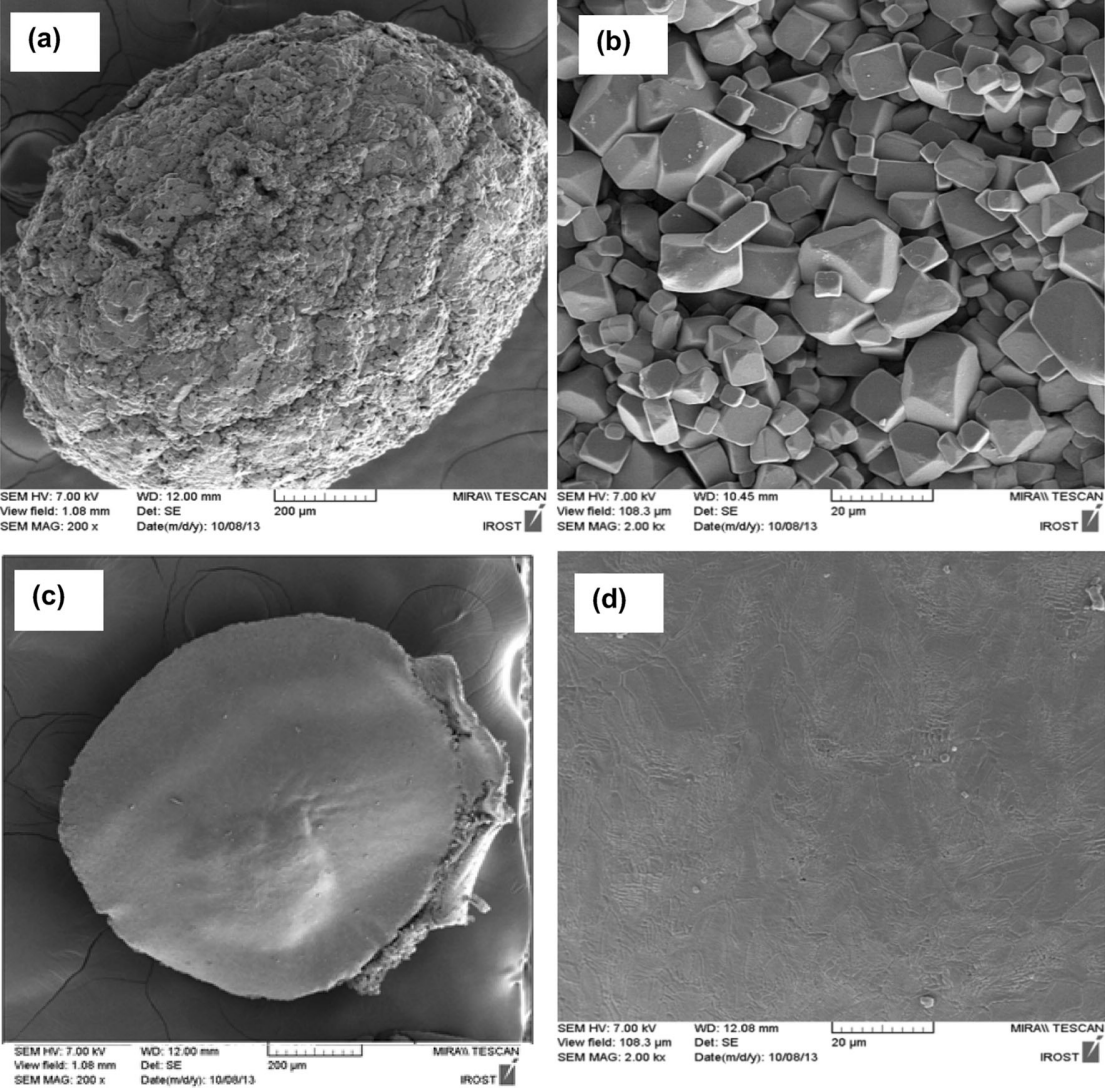
Representative SEM microphotographs of **a** alginate ×200, **b** alginate ×2,000, **c** alginate/dextran–spermine ×200, **d** alginate/dextran–spermine ×2,000 microcapsules

SEM micrographs of microcapsules with corresponding surfaces, with two different magnifications, are shown in Fig. 1a–d. The surface of single-layer alginate microcapsules is coarse as shown in Fig. 1a and b. When dextran–spermine was coated on alginate microcapsules as a second layer, a smooth surface was formed as shown in Fig. 1c and d. It means that the attachment of dextran–spermine decreases the roughness of microcapsules. Polyelectrolyte complexion and ionic bonds between the two polymers resulted in a more smooth structure as well (Madihally and Matthew 1999).

### FTIR analysis

**Fig. 2:**
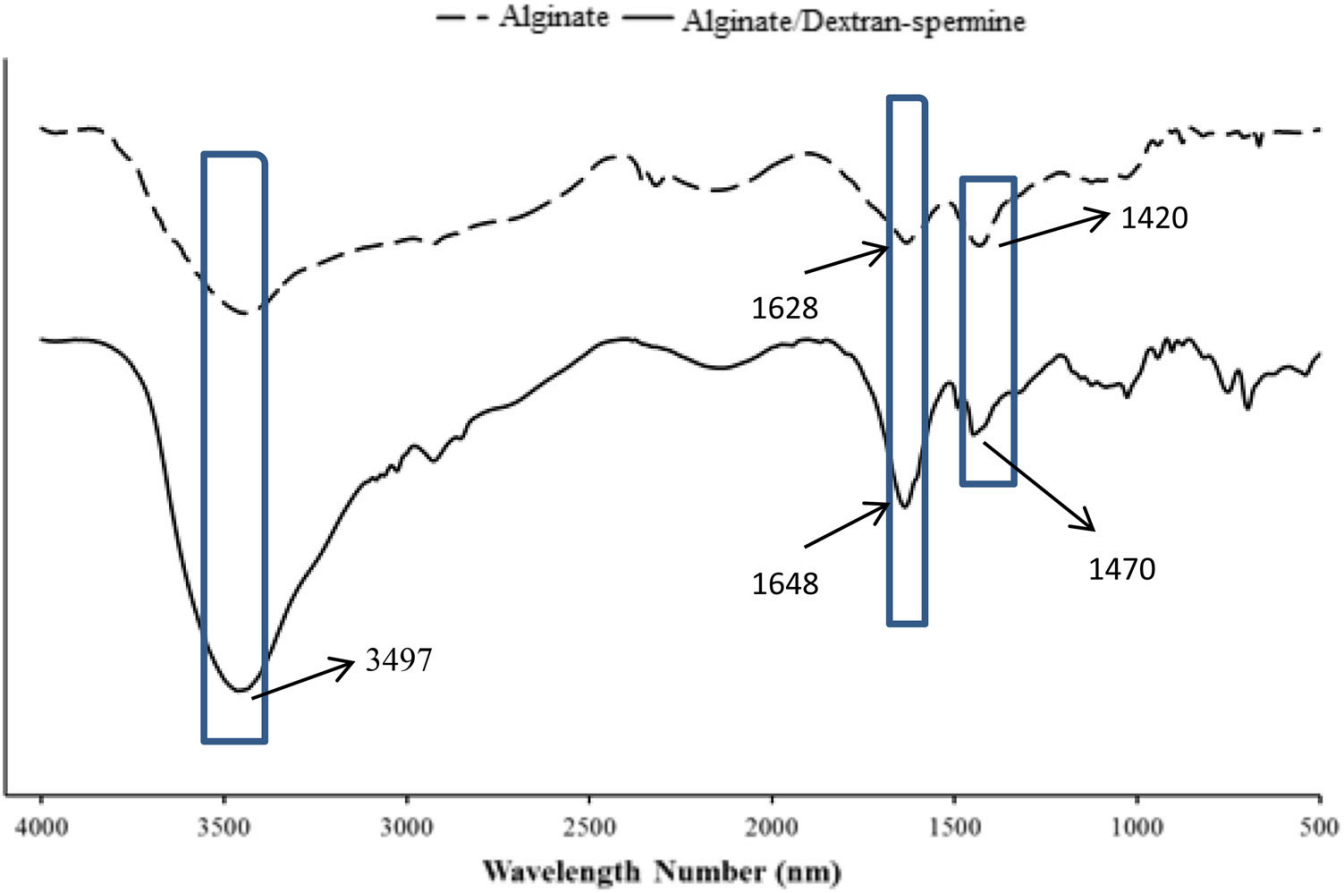
Fourier transform infrared (FTIR) spectra of alginate and alginate-crosslinked dextran–spermine microcapsules

Alginate is an anionic polysaccharide, which forms a polyelectrolyte complex upon interaction with dextran–spermine as a cationic polysaccharide. This special ionic complexion occurs due to the ionic (electrostatic) attraction between the NH_3_^+^ groups of dextran–spermine and COO^**−**^ groups of alginate (Chung et al. 2002). As shown in FTIR diagram, the hydroxyl (OH) vibration bonds and the stretching amino group resulted in a peak at 3497 cm^−1^. The pick in this range became wider after crosslinking with dextran–spermine. The observed peak at 1628 cm^−1^ for alginate switched to 1648 cm^−1^ because of the presence of amino II group. Besides, a peak around 1420 cm^−1^ for alginate microcapsules moved to the left at 1470 cm^−1^ for alginate/dextran–spermine microcapsules, which clearly shows the interaction between NH_3_^+^ in dextran–spermine and COO^−^ in alginate (Fig. 2).

### Pentoxifylline release behavior

**Fig. 3:**
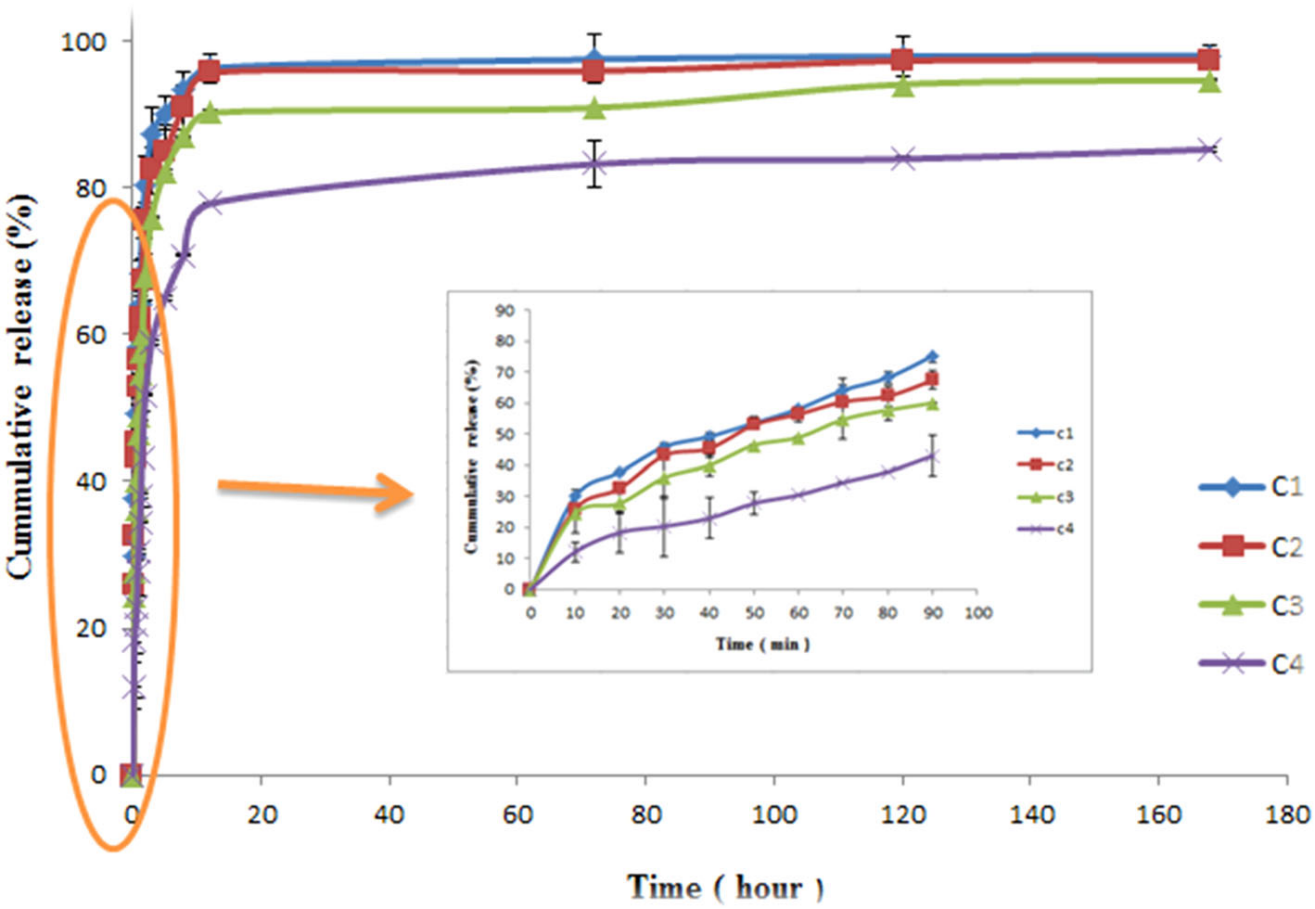
The pentoxifylline release profile of alginate/dextran–spermine microcapsules. *C1* 100 µg/mL, *C2* 125 µg/mL, *C3* 200 µg/mL, *C4* 400 µg/mL

PTX is a hydrophilic drug which can release easily from porous microcapsules. The cumulative release of PTX at four different concentrations was investigated. The samples were taken out of the release medium at specific but different time intervals. Throughout the first 90 min, the samplings were performed with 10-min interval. Then, samples were taken out at 0.5, 1, 2, 3 and 4 h after the previous one until 12 h. At the second stage, at three different time points of 3, 5 and 7 days, the samples were analyzed. As shown in Fig. 3, the rate of drug release decreased by increasing the initial pentoxifylline concentration in polymer solution to be used for preparation of microcapsules. Slow release with less burst effect which is more desirable occurred at 400 µg/mL concentration of pentoxifylline in polymer solution.

### Viability of isolated islets

After islets isolation, they were mixed with AO and PI and viability of them was tested by fluorescence microscopy. The green color indicates the viability of isolated islets.

### Viability of encapsulated and co-encapsulated islets co-cultured with lymphocytes

**Fig. 4:**
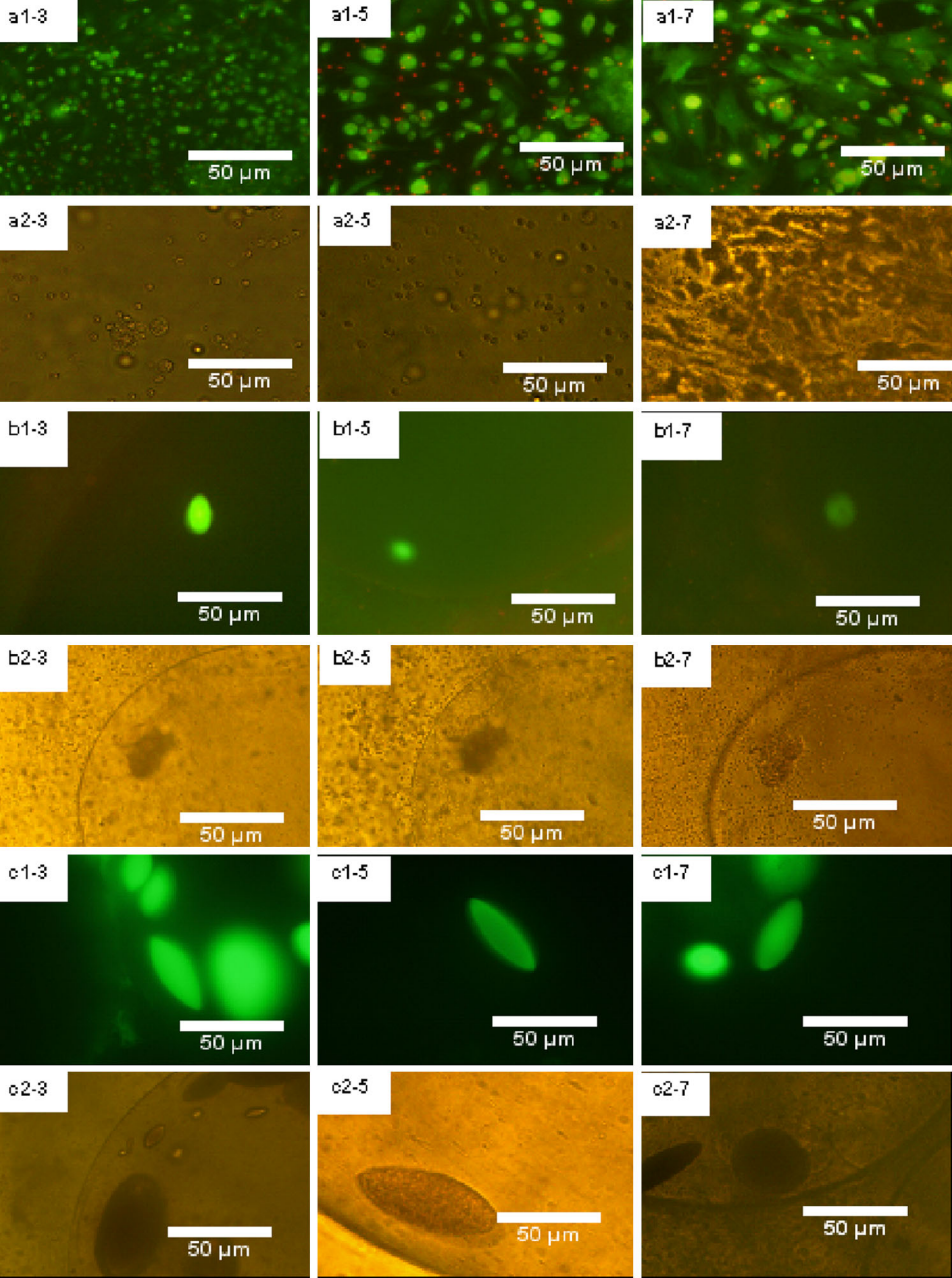
Viability of Langerhans islets using AO/PI assay: intact islets co-cultured with lymphocytes (**a**), encapsulated islets co-cultured with lymphocytes (**b**), co-encapsulated islets co-cultured with lymphocytes (**c**); fluorescent (**a1**, **b1**, **c1**) and phase contrast (**a2**, **b2**, **c2**) images of islets

Intact, encapsulated and co-encapsulated islets were co-cultured with lymphocyte solution. The morphology of intact islets did not change after 3 days of exposure to lymphocytes as shown in Fig. 4a1–3 and a2–3. However, after 5 and 7 days of interaction between intact islets and lymphocytes, the number of red dots which shows dead islets rose (Fig. 4a1–5, a1–7). Although, the formation of cloudy space can be easily observed around encapsulated islets, lymphocytes could not penetrate into capsules which was reported in some studies (de Vos et al. 2006). However, the extent of membrane destruction increased after 7 days as shown by fluorescence images in Fig. 4b1–7 and b2–7. On the other hand, for the islets co-encapsulated with PTX and then exposed to lymphocytes solution till 7 days, the external environment was totally transparent, indicating that PTX can easily suppress lymphocytes attack on encapsulated islets. This suppression effect for co-encapsulated islets continued until 7 days of incubation. By co-encapsulation of islets with pentoxifylline (PTX), the aggregation of lymphocytes around microcapsules decreased as shown in Fig. 4c1 and c2. As illustrated in Fig. 4, the attack of lymphocyte on encapsulated islets increased slowly after 5 and 7 days. It can be observed in Fig. 4b and c that encapsulated islets were detected by lymphocytes, and the extent of this attack increased in the absence of PTX as an immunosuppression drug.

**Fig. 5:**
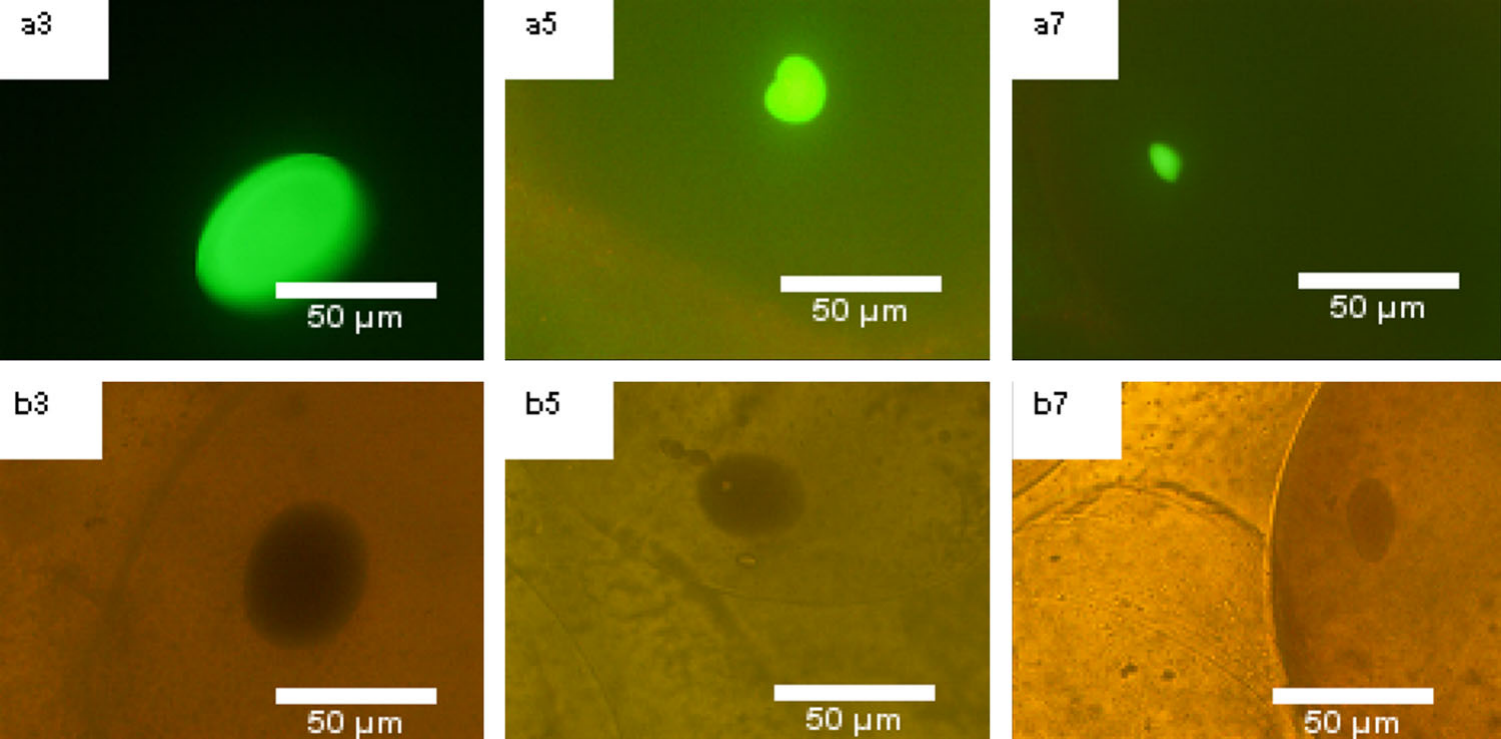
Encapsulated islets co-cultured with lymphocytes in the presence of free PTX after 3, 5 and 7 days: fluorescence (**a**) and phase contrast images (**b**)

In a parallel experiment, encapsulated islets were exposed to lymphocytes in the presence of free PTX in the culture medium. As illustrated in Fig. 5, there was no attack by lymphocytes on encapsulated islets due to immunosuppressive effect of pentoxifylline. It is important to mention that phase contrast images were brought to show attack of lymphocytes since these lymphocytes could not be seen in fluorescent images. Hence, red dots and green circles in fluorescent images indicated dead and viable islets, respectively.

### Effect of pentoxifylline on IL-2 release

**Fig. 6:**
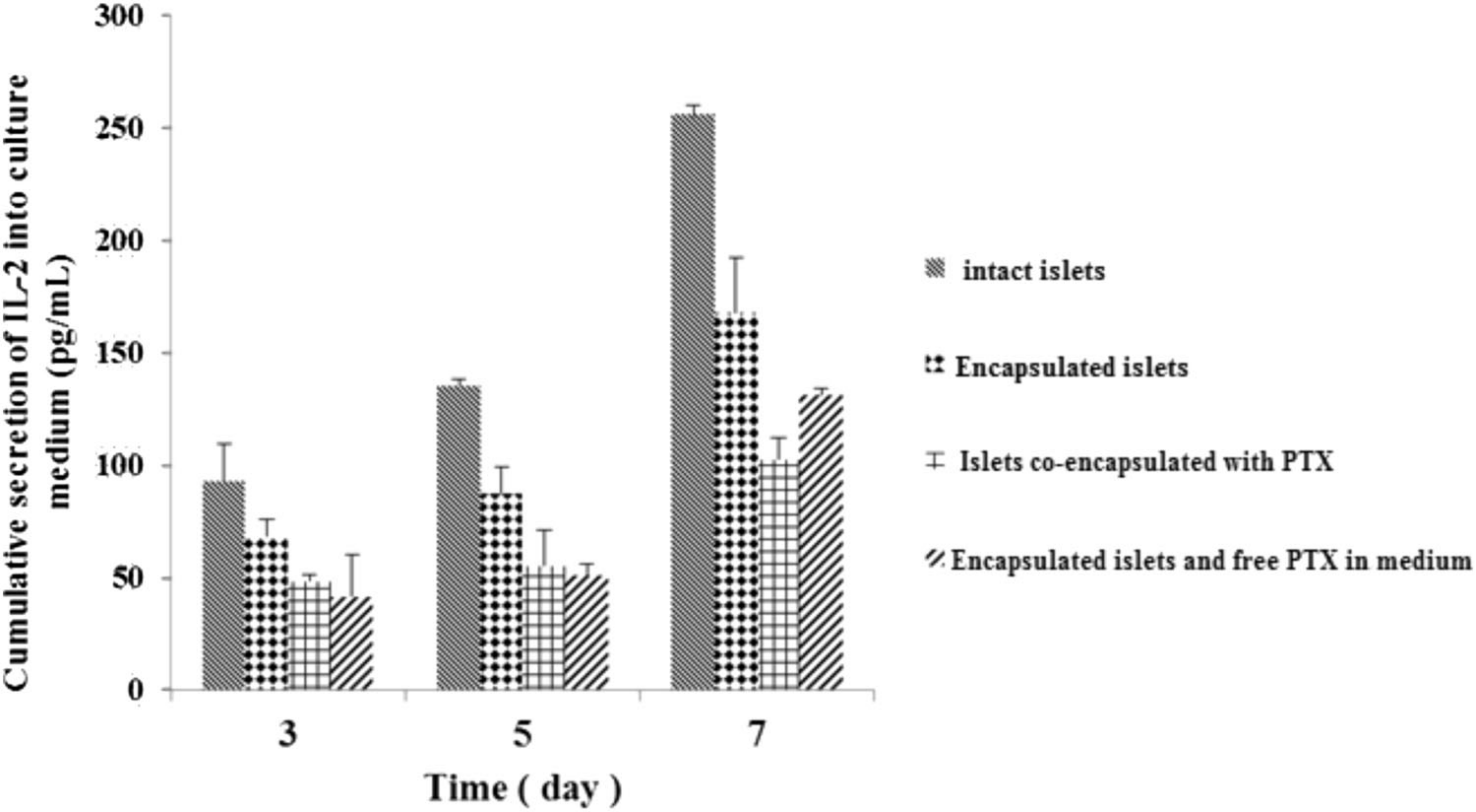
The amount of IL-2 secreted from lymphocytes after 3, 5, and 7 days of co-culturing with lymphocyte (data are presented as mean ± SE, *n* = 4)

Figure 6 shows the amounts of IL-2 secreted from lymphocytes co-cultured with intact, encapsulated and co-encapsulated islets after 3, 5, and 7 days of incubation. Intact islets stimulated lymphocytes more than encapsulated and co-encapsulated ones. Moreover, as an extra control test, the encapsulated islets were cultured in a medium containing free PTX equivalent to the amounts in the co-encapsulated ones (400 µg/mL).

When the intact islets co-cultured with lymphocytes, the amounts of IL-2 secreted from lymphocytes increased from 92.8 ± 16.31 to 135.2 ± 3.2 pg/mL after 3 and 5 days of incubation, respectively, then followed by an increase to 255.8 pg/mL after 7 days of incubation. At the same time, although the amount of IL-2 secretion decreased for encapsulation and co-encapsulation islets, exposure of encapsulated islets to free PTX in the external solution at a concentration of 400 µg/mL led to lower lymphocytes stimulation. This can be attributed to the higher concentration of free PTX in the external solution compared with concentration of PTX released from microcapsules. However, this trend continued up to 5 days of incubation. In fifth day, the amount of IL-2 secretion became nearly equal for both islets co-encapsulated with PTX and encapsulated islets in the presence of free PTX. In seventh day, co-encapsulation with PTX helped to slow releasing of this immunosuppressive drug which prevented IL-2 secretion more effectively (102.4 pg/mL) compared with encapsulated islets in the presence of free PTX in the culture medium (131.6 pg/mL). The effect of slow release of PTX from alginate-based microcapsules to reduce the production of TNF-α and IL-6 by activated macrophage cells has been reported (Lin and Ciou 2010). Besides, these results can be compared with those obtained by PEGylation of islets (Aghajani-Lazarjani et al. 2010) with mPEG-SPA and mPEG-SC as a physical protection. They reported that the cumulative IL-2 secretion against PEGylated islets after 7 days of incubation was about 131.83 and 156.09 for mPEG-SPA and mPEG-SC, respectively. In this study, the accumulated IL-2 secretion against co-encapsulated islets with PTX after 7 days was 102.4 pg/mL which is lower than the values in the aforementioned study that PEGylated islets were exposed to lymphocytes at the same concentration of the present study.

## Conclusion

The results of the present study indicate that double-layer microcapsules of alginate/dextran–spermine are promising candidates for encapsulation of pancreatic islets and their protection against the immune system. Co-encapsulation of the islets with pentoxifylline (PTX) as an anti-inflammatory drug increased the resistance of islets against lymphocytes. Free PTX in the culture medium was effective for immunosuppression as well. This can be attributed to higher concentration of free PTX with respect to PTX released from microcapsules into the culture medium. But, as a whole, co-encapsulation of pancreatic islets with PTX overtakes the encapsulated islets exposed to free PTX in reducing IL-2 secretion when co-cultured with lymphocytes at longer times, due to the slow release of PTX in the medium. Further studies on in vivo transplantation of co-encapsulated islets and functionality of pancreatic islets are required to evaluate the response of the immune system to cell therapy in diabetes mellitus.
